# Empowering Older Patients in Advance Care Planning: Evaluating MyCare™ as a Decision Support Tool

**DOI:** 10.1111/hex.70545

**Published:** 2026-01-30

**Authors:** Alixe Ménard, Yamini Singh, Christina Yin, Sydney Ruller, Lauren Konikoff, Jackie Kierulf, Carol Bennett, Douglas Manuel, Shane Kirkham, Daniel Kobewka

**Affiliations:** ^1^ Ottawa Hospital Research Institute Ottawa Ontario Canada; ^2^ Bruyère Health Research Institute Ottawa Ontario Canada; ^3^ Department of Medicine University of Ottawa Ottawa Ontario Canada; ^4^ Department of Family Medicine University of Ottawa Ottawa Ontario Canada

**Keywords:** advance care planning, autonomy, goals of care, palliative care, shared decision‐making

## Abstract

**Background:**

Older adults with serious illness often face treatment decisions shaped by personal values, yet recognising and expressing those values in ways that guide decision‐making can be challenging. This study evaluated the acceptability, usability and effectiveness of MyCare, a digital values clarification tool designed to help older adults identify and communicate their care preferences to substitute decision‐makers, healthcare providers and support persons.

**Methods:**

This convergent mixed‐methods study explored older adults' experiences with MyCare. Acceptability was assessed using the Theoretical Framework of Acceptability survey and qualitative interviews examining perceptions of the tool's relevance and ease of use. Usability was measured with the System Usability Scale and participant feedback on navigation and clarity. Potential effectiveness was evaluated based on self‐reported ability to articulate and discuss care preferences. Semi‐structured interviews explored these outcomes in depth and were analysed using thematic analysis.

**Results:**

A total of 15 participants (mean age = 83.5 years, SD = 7.6) were surveyed and interviewed between November 2024 and January 2025, including five dyads (one son and mother, three husband‐wife pairs, and two sisters). Key themes identified included: (1) navigating autonomy and delegation in end‐of‐life decision‐making, (2) fragmented communication, (3) influence of lived and observed experiences on care decisions, (4) fears around acceptable and unacceptable quality of life, and (5) using MyCare to promote proactive care planning. Participants reported moderate electronic health literacy, and survey results indicated high usability (SUS mean 83.2/100) and strong acceptability of MyCare, particularly in comfort, perceived value and ease of use.

**Conclusions:**

MyCare demonstrated high acceptability and usability, with participants finding it effective in clarifying and communicating care preferences. It facilitated discussions about quality of life and decision‐making, though challenges remain in addressing prognostic uncertainty and aligning preferences with realistic care goals. Future research should refine the tool to enhance its integration into routine healthcare settings.

**Patient or Public Contribution:**

A patient partner was involved from the outset of this study as a co‐author and co‐decision‐maker in the design and development of MyCare. Their lived experience as the spouse of someone with a serious illness informed the structure and priorities of the tool to ensure its relevance to patient needs. Following this, we engaged additional patients as research participants to provide feedback on usability and functionality. Their insights are being used to refine and adapt MyCare to maximise its impact for future patients.

**Abbreviations**
**eHLA**
: E‐health literacy assessment
**mHOMR**
: Modified Hospitalized‐Patient One‐Year Mortality Risk
**QoL**
: quality of life
SD: standard deviation
**SUS**
: System Usability Scale
**TFA**
: Theoretical Framework of Acceptability

## 1 Background

Older adults living with a serious illness are often faced with medical treatment decisions that require them to weigh the value of potential outcomes [1]. For example, surgery for colon cancer may prolong life but cause discomfort and functional decline [2]. Conversely, choosing not to have surgery may maintain independence and comfort but comes with a high risk of disease progression and eventual death [2]. Thus, the right decision for individual patients depends on the importance they attribute to longevity, comfort and function [3]. Given the complex nature of decision‐making in serious illness, advance care planning (ACP) provides a structured process that enables individuals to define their goals and preferences for future medical treatment and to communicate these preferences to their family and care team so that their wishes are respected [4, 5].

ACP conversations between primary healthcare providers, patients with advanced illnesses, and their families remain challenging [6, 7, 8]. Physicians often prioritise treatment‐focused discussions, while patients may feel unprepared and unaware of options centred on symptom relief and quality of life (QoL) [6, 7, 8]. Meanwhile, family members may experience stress and uncertainty when asked to participate in medical decisions without a clear understanding of their relative's wishes [6, 7, 8]. Further, healthcare providers across primary and secondary settings report low levels of knowledge and confidence in discussing ACP [9]. Many are uncertain about when to initiate these conversations, fear damaging the patient‐provider relationship, view it as a low priority, or feel constrained by a lack of time to engage in meaningful discussions with patients [9]. Additionally, ACP has not consistently improved alignment between patient preferences and their received treatment [10]. For patients, a key challenge is the need to weigh abstract trade‐offs between comfort, function and longevity for hypothetical future decisions [11].

Given these challenges, effective values clarification (i.e., the process of articulating and prioritizing personal values) benefits from the clarity of real‐time decision‐making, allowing patients to weigh specific options with concrete risks and benefits rather than relying on abstract scenarios [12]. While several standardized tools have been shown to effectively prepare patients for in‐the‐moment decision‐making, reducing anxiety and depression while improving bereavement outcomes for family members [13, 14, 15], a gap remains in providing a scalable, structured solution that seamlessly facilitates communication between patients, their family and their care. Notably, widely used ACP tools such as the Serious Illness Conversation Guide primarily focus on equipping healthcare providers with structured approaches to initiate and navigate ACP conversations, with less emphasis on preparing patients in advance to participate in these conversations [14]. Similarly, the PREPARE tool supports patients in reflecting on values and goals, such as preferences for longevity or comfort, but offers more limited support for translating these values into preferences for specific situational medical decisions (e.g., tolerance for prolonged hospitalization or intensive treatments in exchange for modest life extension) [15].

To address this gap, we designed a tool called MyCare. MyCare helps patients express and communicate their most important values when making health decisions, ensuring that this information is shared efficiently with their care team and loved ones [16]. It was designed to address several barriers to care planning conversations, such as limited time and rare expertise in ACP [17]. By emphasising concrete trade‐offs and goals, MyCare provides a clear framework to guide discussions between patients, healthcare providers and care partners. Our study aimed to evaluate the acceptability, usability and effectiveness of MyCare for older adults and assess whether it helps them communicate their care wishes and goals to substitute decision‐makers (SDMs), healthcare providers and family members.

## 2 Methods

### 2.1 A New Tool to Clarify Values and Goals

MyCare was developed to guide older adults to engage in ACP activities by helping them (1) prepare for difficult medical treatment decisions; (2) select a person who could make decisions for them if they were unable to make decisions for themselves; and (3) talk to their family, friends and healthcare team about what is most important, by ensuring their wishes are known. MyCare integrates elements of the Serious Illness Conversation Guide and the Graphic Values History Tool into a single, user‐friendly format tailored for older adults in long‐term care and community settings [13, 14]. It is designed for use both independently by older adults and care partners and interactively with healthcare providers, making it adaptable across contexts. The use of plain language, short manageable sections and visual supports also makes it accessible for individuals of varying literacy levels. MyCare is currently delivered in a secure web‐based format, with efforts underway to develop a dedicated mobile application. It can be accessed on computers, tablets and smartphones. The tool is made up of four parts: (1) About You, (2) Goals of Care, (3) Difficult Medical Treatments and (4) Critical Abilities. The *About You* section gathers information about the individual's health, strengths, fears and abilities. Users can choose from predefined options or provide personalised responses. The *Goals of Care* section is based on the Serious Illness Conversation Guide and helps identify personal goals and preferences for care if health declines, addressing comfort, communication with loved ones, peace, independence and meaningful activities [14]. It expands upon the original guide by incorporating structured prompts for concerns, sources of strength and non‐medical priorities, making the reflection process more holistic. The *Difficult Medical Treatments* section invites users to reflect on their willingness to undergo treatments that might extend life but may result in increasing physical decline (e.g., a long hospital stay). These scenarios are presented visually and in plain language to improve accessibility for individuals with varying health literacy. The *Critical Abilities* section is based on the Graphic Values History Tool [13]. This section focuses on abilities that could influence decisions about life‐extending treatments, such as feeding, washing, breathing, decision‐making, communication and recognising loved ones. MyCare enhances this approach by linking each ability to potential treatment implications and explicitly reaffirming that symptom management (e.g., pain and nausea) will always be provided, regardless of choices about life‐prolonging care.

### 2.2 Study Design

We employed a convergent mixed‐methods design to assess the usability and acceptability of MyCare among older adults. Convergent mixed methods is a research design in which quantitative and qualitative data are collected concurrently, analysed separately and then merged during data interpretation, allowing for a more comprehensive understanding of the study objective [18, 19]. Quantitative data were collected using three validated measures: (1) the E‐Health Literacy Assessment (eHLA) [20], (2) System Usability Scale (SUS) [21] and (3) Theoretical Framework of Acceptability (TFA) [22]. The eHLA is a validated tool that assesses self‐reported computer familiarity, confidence and incentive to engage with technology [20]. *Familiarity* is evaluated on a 4‐point scale, ranging from ‘not at all familiar (1)’ to ‘completely familiar (4)’, and includes aspects such as keyboard use, navigating settings, operating systems and usernames [20]. *Computer confidence and incentive* to use technology are measured in relation to general computer use, touchscreen interaction and finding information online [20]. The SUS is a 10‐item Likert scale that provides a global assessment of a tool's usability based on users' subjective experiences [21]. This scale evaluates ease of use, complexity, confidence and the need for technical support, with higher scores indicating better usability [21]. Finally, the TFA aims to understand how individuals perceive the desirability and appropriateness of a tool, considering factors like ease of use, perceived benefits and alignment with personal or societal values [22]. Qualitative data were subsequently gathered through semi‐structured interviews, focusing on MyCare usability, its potential impact on confidence in care discussions, ACP awareness and recommended tool improvements. The semi‐structured interview guide is presented in Appendix 1.

### 2.3 Study Recruitment

Eligible participants were identified through electronic medical records at The Ottawa Hospital (TOH). Eligible patients had: (1) a Modified Hospitalized‐Patient One‐Year Mortality Risk (mHOMR) score of ≥ 20, indicating a high risk of mortality within the next year and a potential need for early palliative care interventions [23]; (2) age ≥ 65; and (3) receipt of care at TOH within the past 5 years (2019–2024), (4) were fluent in English and (5) able to consent for themselves. Participants were contacted and consented by phone. Study personnel then scheduled an interview at a time and location convenient to the participant, either at their own home or at the hospital. To support the evaluation process, participants were encouraged to involve an SDM, if desired or needed.

### 2.4 Sample Size

We recruited 15 participants, with the sample size determined by data saturation, that is, the point at which no new themes or insights emerged from additional interviews [24]. Due to the sensitive nature of the study, which addressed topics such as difficult treatment decisions and end‐of‐life care, we documented the outcomes of all patient contacts, including reasons for exclusion or unwillingness to participate. Of the 95 patients contacted, 15 agreed to participate, 29 had disconnected or non‐functional phone numbers, 12 requested time to consider participation and follow‐up, 7 declined due to a lack of interest in discussing death and ACP, and 32 deemed themselves not well enough to participate.

#### 2.4.1 Positionality

As researchers committed to improving communication and decision‐making in ageing and healthcare, we recognise that our perspectives shape the development and evaluation of MyCare. Our backgrounds in ageing, palliative care and patient‐centred care informed both the design of the tool and our approach to exploring its usability and acceptability among older adults. We acknowledge that our belief in the importance of ACP may influence our interpretation of participants' experiences. To mitigate potential biases, we employed a mixed‐methods approach, integrating both qualitative and quantitative data to support a comprehensive and balanced evaluation of MyCare. Additionally, we remained attentive to participants' perspectives throughout data collection and analysis, ensuring their voices guided our interpretation of MyCare's impact.

### 2.5 Patient Partner Involvement

A patient partner, whose husband was living with a serious illness, collaborated with the MyCare development team through bi‐weekly meetings during the tool's development. She provided guidance from the initial planning phase through each draft and also recorded the introductory video outlining what patients can expect when using MyCare.

#### 2.5.1 Data Collection

All data were collected in person. Participants first completed a demographic questionnaire, followed by the eHLA. Questions were posed by the researcher and entered directly into the Research Electronic Data Capture (REDCap) system for secure storage and analysis [25]. Next, they engaged with MyCare using a TOH‐provided iPad, navigating the tool aloud, and asked to verbalise their thought processes. Researcher memoing (i.e., clarifying one's thinking and tracking the development of the data through handwritten notes) [25] was conducted throughout the MyCare think‐aloud process, which provided valuable insights into participants' thought processes. MyCare took approximately 15 minutes to complete. This was followed by a semi‐structured interview, after which participants completed the TFA and SUS. All participants completed every survey item, leading to no missing data. All quantitative data were entered into the REDCap system for secure storage and analysis [26]. Our convergent approach allowed us to examine participants' experiences with MyCare and their engagement in ACP before using the tool. Employing mixed methods during instrument evaluation enhanced credibility by assessing appropriateness through both qualitative and quantitative data [27]. All sessions were audio‐recorded, lasted approximately 90 minutes, and were conducted by two researchers (A.M. and Y.S.) to ensure consistency. This study was approved by the Ottawa Health Science Network Research Ethics Board (protocol # 20240464‐01H).

#### 2.5.2 Data Analysis

Quantitative data were analysed using descriptive statistics to summarise the demographic characteristics of the participants and the scores from the eHLA, SUS and TFA. Specifically, means and standard deviations (SDs) were calculated for each of these measures to provide an overview of participants' familiarity with technology, tool usability and perceptions of MyCare's acceptability. This approach allowed us to quantify and assess the overall trends and variability in participants' responses.

For our qualitative data analysis, all semi‐structured interviews were transcribed verbatim. Each transcript was reviewed by a research team member (A.M. or Y.S.) to ensure consistency with the audio files and encourage familiarisation with the data. Identifying information was removed from interview transcripts, and participants were assigned unique identifiers to preserve anonymity. Two researchers (A.M. and Y.S.) collaboratively coded three transcripts, allowing for the development of a consistent coding framework. In alignment with Braun and Clarke's thematic analysis [28], initial codes were created by identifying key phrases, concepts or observations that aligned with the study's objectives, particularly those related to usability, ACP and confidence in healthcare discussions. The resulting codebook is provided in Appendix 2. Following this, each researcher (A.M. and Y.S.) independently coded the remaining transcripts. Codes were then grouped into potential themes that reflected patterns in the data [28]. These themes were reviewed and refined in relation to the entire dataset [28]. Discrepancies in coding were resolved through ongoing team discussions to reach consensus [28]. To enable a thorough analysis, we employed NVivo software to facilitate our inductive thematic analysis [29]. The avoidance of a predetermined codebook allowed us to identify and explore patterns and themes in the data without preconceived notions or expectations [29]. The combination of inductive coding, regular discussions and memoing strengthened the depth and rigour of our qualitative analysis [30].

## 3 Results

### 3.1 Participant Demographics

Of the 15 interviews, five were conducted with dyads. Most dyads were spouses (*n* = 3), followed by sisters (*n* = 1) and a mother‐son pair (*n* = 1). In three cases, only one member of the dyad met the inclusion criteria and consented to participate; the accompanying person, who did not have a serious illness, attended the interview in a supportive role but was not counted as a participant. In the remaining two dyads (a husband‐wife pair and two sisters), both individuals met the inclusion criteria (i.e., a serious illness, age ≥ 65 and receipt of hospital care within the past 5 years), provided consent and were counted as separate participants. The mean age of participants was 83.5 years (SD = 7.56). Sex distribution was nearly equal, with 53.3% identifying as women and 46.6% as men. Most participants lived in private residences (80%), while 20.0% resided in a retirement residence. The majority (93.3%) identified as white and reported Catholic affiliation (60.0%). Educational attainment was high, with 86.7% having some university education, including nearly one‐third holding a graduate degree. In terms of annual household income, 58.3% reported an annual household income between $35,000 and $49,999.

### 3.2 Electronic Health Literacy Assessments

Participants demonstrated moderate familiarity and confidence with digital tools, whereby a score of 1 indicates ‘Not familiar at all’ and a score of 4 indicates ‘completely familiar’. The mean out of 4 (SD) familiarity scores were as follows: keyboards (*M* = 3.2, SD = 1.0), system settings 2.5 (1.3), operating systems 2.5 (1.2) and usernames 2.6 (1.3). Confidence in using these tools varied across tasks: general computer use 2.5 (0.9), touchscreen interactions 2.5 (1.3) and finding information online 2.9 (1.1). Overall, participants displayed moderate confidence and low incentive to engage with digital tools, with mean scores for general computer use 2.9 (1.3), touchscreen use 2.7 (1.2) and finding information online 3.0 (1.2).

### 3.3 SUS

**Figure 1:**
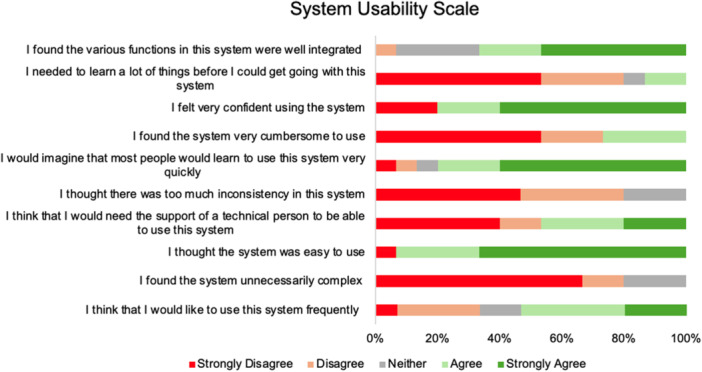
SUS item responses for MyCare.

Participants' responses on the SUS highlighted generally positive usability for MyCare, yielding an overall score of 83.2/100 (SD = 11.5), suggesting a high level of usability, with participants indicating strong agreement with the tool's ease of use and confidence in learning to use it quickly. Figure 1 illustrates the distribution of responses for each SUS item, highlighting consistently high ratings on ease of use, confidence, learnability and low levels of perceived complexity or inconsistency.

### 3.4 Acceptability

**Figure 2:**
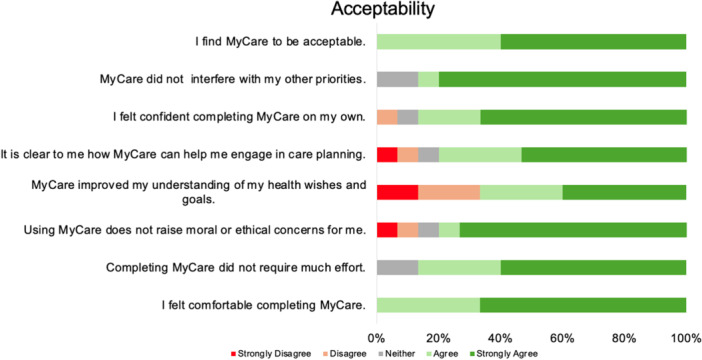
TFA item responses for MyCare.

Participants' responses on the TFA highlighted generally positive perceptions of MyCare, with strong endorsement across key domains of acceptability. Most participants indicated feeling comfortable using the tool and found it aligned with their personal values and required little effort to complete. Participants also expressed confidence in their ability to engage with MyCare and reported that it did not interfere with other priorities. Figure 2 illustrates the distribution of responses for each TFA item, highlighting high ratings on affective attitude, ethicality, self‐efficacy and opportunity costs, along with generally strong perceptions of the tool's effectiveness and acceptability. Appendix 3 presents a summary of the eHLA, SUS and TFA results.

### 3.5 Themes

A total of five themes resulting from the integration of the quantitative and qualitative data were identified through thematic analysis. These themes included: (1) navigating autonomy and delegation at the end of life, (2) fragmented communication, (3) influence of lived and observed experiences on care decisions, (4) fears around acceptable and unacceptable QoL, and (5) using MyCare to promote proactive care planning.

#### 3.5.1 Theme 1: Navigating Autonomy and Delegation at End of Life

Participants experienced a tension between maintaining autonomy in care decisions and relying on SDMs or powers of attorney (POAs) when they could no longer advocate for themselves. As one participant explained, ‘You have to make sure that the other person understands exactly where you're coming from’ (MM03, age 80). Ensuring that SDMs or POAs fully understood their values was essential. Many participants emphasised the importance of written documentation to avoid misunderstandings, as one participant told her daughter: ‘I would like you to write it down just in case I change my mind’ (MF04, age 80). While participants valued family‐led decision‐making, they also acknowledged the emotional burden these decisions could place on loved ones, especially when not planned in advance of an acute health event. One participant remarked, ‘It [family‐driven decision‐making] puts an enormous amount of stress on everybody that's doing the looking after’ (MF11, age 90), while another worried, ‘I don't think I would give them the questions and my answers [from MyCare] for fear that it would cause them anxiety. I mean, they got their own families, they got their own issues' (MM06, age 79). This interplay between maintaining autonomy, delegation to loved ones and mitigating the impact on family members highlighted the complex dynamics of end‐of‐life planning. One participant offered a piece of advice on how he would broach the topic of ACP and substitute decision‐making with his loved ones:

There's no magic pill. Death is a part of life. These are some options. You don't have to follow any of it. Try and keep in mind it's your responsibility, as much as you want to, to perform that role but don't get immersed in all this stuff. Just say to the people you love, I love you. That's important. Remember the human side of all this.

MM05, age 86

Ultimately, participants underscored the importance of balancing practical decision‐making with the human side of end‐of‐life planning, ensuring that love, trust and clarity guided the process for both them and their families.

#### 3.5.2 Theme 2: Fragmented Communication

When asked about their familiarity with ACP or future planning, many participants referenced having a do‐not‐resuscitate order but were generally unable to describe ACP beyond this. A recurring theme in the data was that there are systemic barriers to effective communication of prognosis and the integration of ACP within healthcare settings. Participants emphasised that healthcare providers often failed to convey the serious nature of a patient's illness or proximity to death in a clear and empathetic manner. One participant said:

I knew what DNR [Do Not Resuscitate] meant, and I was shocked to hear it. I was just shocked. Ooh, am I really that seriously ill? Because I know you need it when you go into the hospital. I knew that in the back of my mind. But to be faced with it like that. I think had the approach been a little different, I would not have been so shocked. I wouldn't have been frightened as I was. I thought I was dying imminently.

MF04, age 80

This lack of communication of a patient's prognosis, marked by a lack of context, clarity or sensitivity, left some participants unaware of the gravity of their conditions, resulting in missed opportunities for meaningful discussions about ACP. These challenges were further compounded by staffing shortages and the lack of continuity of care, particularly in emergency departments where multiple physicians and nurses may care for a single patient. One participant described this deficiency as follows:

You have to tell the triage nurse what's wrong and all this stuff. Then, you have to tell the emergency nurse what's wrong. Then, you have to tell the doctor what's wrong. Then, you have to tell the resident what's wrong, and you're repeating the same story over and over again. I don't think that all of them are going to sit down and read this [MyCare ACP results].

MF09, age 81

Inconsistent or incomplete communication across care teams often resulted in mixed messages or a lack of crucial information regarding prognosis and ACP. Many participants expressed a preference for their primary healthcare provider to lead discussions about their care preferences. However, the shortage of primary healthcare providers meant that some participants had not yet established a trusting relationship with their new healthcare provider.

I'm on my third doctor. He is thorough, but we haven't gotten to the point of discussing the fact that maybe my days are numbered. That has not come up, I guess, because there hasn't really been any reason despite the [lung cancer] diagnosis.

MM07, age 78

The repeated retelling of health histories to different or new providers further underscored inefficiencies in the system and created frustration for patients who depended on their care providers to broach the topic of end of life. This gap created space for a tool like MyCare to capture and share vital information across providers.

My doctor could say, listen, you're close to 80, right? Things can change fairly rapidly, et cetera. So, here's something for you to consider [the MyCare tool]. It would be introduced in such a way as to say, hey, this is just—this is planning. Then, it would go from my family doctor and it would come to the attending physician at the hospital.

MM07, 78

#### 3.5.3 Theme 3: Influence of Lived and Observed Experiences on Care Decisions

Participants frequently described how their personal experiences or those of loved ones significantly shaped their decisions regarding future care. Observing the challenges faced by parents, friends or neighbours, particularly negative experiences with nursing homes, served as a powerful motivator for planning and decision‐making.

I looked after an old lady who lived across the road and became her executor. We had to move her out of her home and into a nursing home and from there I had to take her to all kinds of appointments that she had about her health. (…) So she sort of became a blank sheet for me. Because if it was concerning her now, it was going to concern me pretty soon. I learned from her.

MF11, age 90

For some participants, witnessing the physical and emotional toll of illnesses such as stroke or the loss of autonomy in care settings prompted reflections on what they would prefer for themselves. These experiences often became a blueprint for participants, informing their preferences and instilling a desire to avoid similar outcomes. Many participants, even those in advanced age (90+), continued to make medical decisions based on what their parents had experienced in late life, such as needing assistance with bathing or feeding in a care setting. These formative memories appeared to shape their perceptions of dignity, independence and acceptable levels of care, underscoring the lasting influence of intergenerational experiences on end‐of‐life decision‐making.

My mother had a stroke, and it was 4 years in a nursing home without speech and with having to be fed and all the rest of it. So, we definitely know we don't want that.

MM02, age 89

This reliance on observed experiences underscored the deeply personal and emotional nature of care planning, as participants used these narratives to anticipate and articulate their own values and priorities.

One of my sons had a massive stroke, and he was on life support for several days before his wife gave the permission to take him off. So, things like that, I wouldn't want to be like that.

MM08, age 97

However, this also highlighted how decisions about care are often shaped more by anecdotal evidence than by discussions with healthcare providers, raising questions about how the healthcare system can better support proactive, informed decision‐making grounded in both personal values and medical guidance.

#### 3.5.4 Theme 4: Fears Around Acceptable and Unacceptable QoL

Participants articulated deeply personal definitions of acceptable and unacceptable QoL, which guided their reflections on treatment preferences, living arrangements and end‐of‐life decisions. Acceptable QoL often revolved around maintaining cognitive function and the ability to communicate and enjoying meaningful interactions with loved ones.

You want the treatment, no matter what it is, if you're going to end up with quality of life at the end; if you're going to be able to interact with your wife, talk to your wife, enjoy the things that she enjoys, you know?

MM03, age 80

For many, the capacity to live independently at home was pivotal to QoL. ‘Our plan for acceptable quality of life is to stay here [at home] being self‐sustaining as long as possible’ (MM07, 78).

Other participants described QoL and a life worth sustaining as being able to continue to engage with family, even if they required assistance with activities of daily living:

If my mind is there and I'm quite aware of my environment, I would accept somebody feeding me. I would accept somebody, as much as the toileting would bother me, yes, because that's still life where I have that much longer to talk to my children, you know, to see my family and new grandchildren. That's important, especially when you get older.

MF04, 80

Conversely, unacceptable QoL was closely tied to fears of prolonged suffering, loss of autonomy and dependency on institutional care. The notion of lingering in a state of severe physical or cognitive decline was particularly distressing, with some participants expressing a preference for hastened death over enduring such conditions. For instance, participants highlighted their fear of being unable to think, communicate or maintain any semblance of independence as central to their aversion to certain care outcomes.

And I think to myself, I could accept a lot of things as long as I could still think. As long as I can see and I'm cognitive, I'd like to be able to hold something so I can use the iPad. But, you know, if it meant somebody else doing it, I don't know that I'd want to be like Stephen Hawking and have to use my mouth and things. I mean, I admire him, but I don't think it's for me.

MF09, age 81

Religious and moral considerations also emerged, influencing participants' perspectives on medical assistance in dying (MAiD) and their tolerance for suffering. These reflections revealed an internal struggle between personal values, religious beliefs and the desire to avoid pain and indignity at the end of life.

I was telling the doctor that I don't do pain very well. I'm afraid of pain. I had said, I'm Catholic and they don't really believe in MAiD, but when you're in agony, do you care?

MF09, age 81

Overall, participants' perceptions of QoL were shaped by a complex interplay of personal experiences, societal norms and existential fears. Their views on what constituted an acceptable or unacceptable QoL were often influenced by their willingness to accept assistance in exchange for the loss of bodily abilities, highlighting the nuanced balance between independence and care.

#### 3.5.5 Theme 5: Using MyCare to Promote Proactive Care Planning

Participants viewed the MyCare platform as a tool that facilitated proactive reflection on care preferences, helping individuals think ahead about their future care needs rather than confronting these questions in the moment. Several participants emphasised the value of having time to consider their care preferences and saw it as crucial for reducing stress and ensuring that their wishes are clear to both loved ones and healthcare providers at the time of an acute health event.

Well, it's better to make the decision before something happens. For example, your decision‐making might be clouded by pain or distracted because of something else that has happened. So, when your mind is clear, you should think about it then rather than when it happens.

MF01, age 91

Participants also highlighted MyCare's potential role in breaking down barriers to difficult conversations around end‐of‐life care preferences, enabling families and patients to engage in discussions that might otherwise be avoided. MF01 said, ‘I have a feeling that there are things that my daughter and I won't talk about unless there's something that triggers it and MyCare triggers it’. The platform was seen as both a tool for summarising current health statuses and preferences, as well as a mechanism to prompt individuals to think about their care in a structured manner. As one participant described it, ‘I see it as being a tool that summarises where I'm at and where I want to be’ (MM07, age 78). This utility was especially appreciated for its role in ensuring that participants' care preferences were clearly understood and effectively communicated to their loved ones and healthcare providers. A few participants reflected on the emotional aspect of end‐of‐life care decisions, noting that MyCare could help reduce emotional burden by providing a more organised, clear way to think about care options.

The next time I see my doctor, I'm going to tell him about this advanced care planning tool, and that I've had the opportunity to participate [in this study], but also to learn and to focus the mind, because that's what [MyCare] does, right? I'm sure that's one of the intended purposes is to instead of getting all emotional, is that we have some things to do here. We have to get them down in the right order.

MM07, 78

MyCare was seen as a tool to not only clarify care preferences but also help individuals prepare mentally and emotionally for future decisions. However, there were concerns about the ongoing relevance of the information, with one participant wondering if the responses would remain valid as her husband's health changed over time. ‘Two years down the road, he'd be 99 and a half; is that still relevant?’ (MF09, age 81). This highlighted the need to regularly update care preferences to ensure they remain aligned with a person's evolving health status. MyCare's potential to ease the decision‐making process and ensure that care preferences are communicated clearly was evident to participants, but effective implementation would require further exploration.

## 4 Discussion

Our study assessed the usability, acceptability and potential effectiveness of MyCare, a digital tool designed to support advanced care planning by facilitating communication of care preferences among older adults, their SDMs, healthcare providers and family members. Overall, participants found MyCare user‐friendly and acceptable, though integration into existing care processes remains a challenge.

We identified five key themes that describe participant engagement with ACP using MyCare: (1) navigating autonomy and delegation at end of life (i.e., balancing personal control over decisions with reliance on family members), (2) fragmented communication (i.e., systemic challenges in conveying prognosis clearly and sensitively resulting in fragmented care), (3) influence of lived and observed experiences on care decisions (i.e., how familiar experiences with illness, loss of autonomy and institutional care shaped participants' preferences), (4) fears around acceptable and unacceptable QoL (i.e., how participants evaluated QoL and suffering in relation to autonomy), and (5) using MyCare to promote proactive care planning (i.e., how the platform helped participants reflect on and clarify a person's care preferences in advance of acute health events). Our themes highlight the complexities of end‐of‐life decision‐making, the role of communication in care planning, and the potential for digital tools like MyCare to support proactive engagement in these discussions.

Participants found MyCare generally easy to use, with high ratings for usability as measured by the SUS [21]. MyCare was seen as intuitive, requiring minimal technical support, and most participants reported high levels of comfort using it. These findings align with participants' moderate familiarity with digital tools and their confidence in performing basic digital tasks. However, the moderate levels of digital literacy observed in participants, coupled with relatively low incentives to engage with digital tools, suggest that while MyCare is accessible, further simplification and more personalised engagement strategies would be beneficial. This is particularly important for individuals with lower digital literacy or less motivation to engage with technology [31]. Future iterations of MyCare may benefit from features that explicitly address these barriers, ensuring broader adoption and sustained use [32].

The TFA results revealed high acceptability of MyCare, with participants expressing comfort in using the tool, perceiving it as effective and experiencing minimal interference with other daily priorities. Many participants appreciated that MyCare aligned well with their personal values and care preferences. Participants also viewed MyCare as effective in promoting ACP by helping facilitate discussions between patients, their families and healthcare providers. In contrast to clinician‐focused tools such as the Serious Illness Conversation Guide, which primarily support providers in leading ACP conversations [14], MyCare was perceived as highly usable and acceptable for independent patient use. This patient‐oriented usability may help bridge gaps in ACP engagement by offering a more accessible entry point for older adults with varying levels of health literacy. Although MyCare was seen as a helpful resource for ACP, its full potential may be realised only if it is incorporated into existing healthcare workflows, particularly as participants noted external barriers such as fragmented communication systems and inconsistent ACP facilitation by healthcare providers [11]. Integrating MyCare into routine healthcare interactions, such as medical appointments or community‐based settings, may serve to normalise ACP discussions and encourage earlier engagement with care planning tools [11].

Moreover, the effectiveness of MyCare in improving ACP outcomes was influenced by broader systemic challenges. Participants listed issues such as communication barriers within healthcare settings, the lack of structured guidance from healthcare providers, and the variability in the timing of ACP discussions. These findings resonate with existing literature on ACP, which underscores insufficient time and opportunity, absence of a supportive culture around ACP, fragmented communication systems, and uncertainty regarding who should take the lead in initiating discussions as key barriers to effective ACP [33]. Many participants noted that discussions about end‐of‐life care often defaulted to DNR orders, making it the first topic raised rather than a more holistic conversation about values and goals of care. This narrow framing can be distressing for patients and families, as it reduces ACP to a single decision rather than a broader dialogue about future care preferences [34]. Moreover, while early ACP discussions are encouraged, they are often delayed until a crisis occurs, at which point decision‐making may become rushed and emotionally overwhelming [35]. Another significant barrier is the shortage of family physicians, which creates additional challenges for patients seeking continuity in their care [36]. A longstanding relationship with a family doctor can provide an essential anchor to the healthcare system, allowing for the gradual introduction of ACP discussions within a framework of trust [9]. However, with the growing physician shortage, many older adults do not have a consistent provider who can initiate these conversations and guide them through the decision‐making process [37]. Without a trusted medical professional to facilitate ACP, patients may be left without the necessary support to articulate and document their preferences, increasing reliance on family members who may not be equipped to navigate these discussions [38].

A significant barrier identified in this study was the limited incentive for participants to engage with digital tools, despite their moderate familiarity and confidence with technology. Participants cited factors such as perceived relevance, interest and prior experience with digital tools as key influences on their motivation to use MyCare. These findings highlight the need for tailored strategies to enhance engagement, particularly for individuals with lower digital literacy or less intrinsic interest in technology. One potential approach, suggested by participants, is integrating MyCare into routine medical appointments or community‐based settings, which could help normalise ACP discussions and encourage earlier engagement with the tool. Beyond motivational barriers, MyCare's design must also account for varying levels of digital literacy. Offering both self‐directed and guided options, along with ongoing support from healthcare providers, could enhance both its usability and appeal. Additionally, expanding MyCare's accessibility to include multiple languages and cultural contexts would ensure it meets the needs of a more diverse population.

Notably, participants frequently referenced their own past experiences, as well as those of their parents and other family members, when making decisions about their future care. Even among older adults, reflections on their parents' end‐of‐life experiences played a crucial role in shaping perspectives on autonomy, institutional care and preferred interventions. Personal and vicarious experiences with illness, hospitalisation and long‐term care have been shown to influence perceptions of what constitutes a ‘good’ or ‘bad’ death [39, 40, 41]. Emerging research suggests that such experiences significantly shape preferences: individuals who witness distressing scenarios (e.g., prolonged mechanical ventilation, cognitive decline or institutional isolation) often seek to avoid similar circumstances, whereas those who observe positive care experiences feel reassured about ACP and shared decision‐making [42].

Participants also expressed diverse views on what constitutes an acceptable versus unacceptable QoL, particularly regarding autonomy, cognitive function and physical dependence. Some viewed severe cognitive impairment or total physical dependence as states worse than death, raising ethical concerns about how such beliefs influence ACP decisions. Many ACP discussions attempt to elicit these perspectives through hypothetical scenarios, asking individuals to consider which conditions they would find intolerable [43]. However, the effectiveness of this approach remains debated [11]. While articulating thresholds for acceptable QoL can help guide future care, it may also oversimplify the complexities of real‐world decision‐making, where preferences often evolve over time [11]. Moreover, framing decisions in terms of ‘states worse than death’ risks reinforcing ableist perspectives by positioning disability or dependence as inherently undesirable [44]. A more nuanced approach could encourage individuals to consider values beyond functional ability, such as relationships, dignity and comfort, rather than focusing solely on loss of autonomy or physical capacity [45]. MyCare exemplifies this approach by broadening ACP discussions to include these dimensions.

Digital tools like MyCare have the potential to support ACP by offering a structured framework for discussing and documenting care preferences. One key consideration is determining when and by whom the tool should be introduced. Some participants felt that using MyCare during routine medical appointments could normalise ACP discussions and encourage earlier engagement. Others suggested that community‐based settings, such as senior centres or caregiver support groups, might provide a less clinical and more supportive environment for initiating these conversations. This suggests that successful implementation will depend not only on where and when MyCare is introduced, but also on ensuring appropriate facilitation and integration into routine care, so that conversations about preferences are both timely and person‐centred.

### 4.1 Strengths and Limitations

Our study has several strengths, including the use of a convergent mixed‐methods design, which allowed for a comprehensive evaluation of MyCare by integrating both qualitative insights and quantitative measures [46]. The inclusion of older adults with diverse care experiences, either their own or their relatives (e.g., with palliative care, cancer care and assisted living), provided valuable perspectives on the usability and acceptability of the tool in real‐world settings. Additionally, the incorporation of validated instruments, such as the SUS [21] and the TFA [22], strengthened the assessment of MyCare's effectiveness. Our study benefited from dyadic participation, enabling insights into how MyCare facilitates communication between older adults and their SDMs [47]. However, some limitations should be acknowledged. The study's recruitment was limited to a single site, TOH, which may restrict the generalisability of findings to broader populations, including those receiving care in different healthcare settings or cultural contexts. Additionally, MyCare is currently only available in English, which may exclude non‐English‐speaking individuals and limit the accessibility of the tool for a linguistically diverse population. The sample was predominantly white and consisted of individuals with relatively high levels of education. As a result, the experiences of persons from minority groups or with lower educational attainment may not be fully represented in the findings. Our results are prone to social desirability bias, as participants may have provided responses they believed were expected [48]. Future research should address these limitations by expanding the study population to include more linguistically and culturally diverse groups, incorporating individuals from minority groups, exploring the experiences of those with lower educational attainment, including people living in LTC settings, and examining the long‐term effects of MyCare on decision‐making and care outcomes.

## 5 Conclusion

Our findings highlight the complex interplay between personal experiences, systemic barriers and technological solutions in shaping ACP engagement. MyCare demonstrated strong usability, acceptability and potential effectiveness in promoting ACP and holds promise in facilitating structured conversations and improving documentation of care preferences. Its full impact on end‐of‐life decision‐making will depend on provider engagement, integration into existing healthcare processes, and overcoming barriers related to digital literacy, motivation and engagement. Future research should explore strategies for optimising the timing and delivery of digital ACP tools, as well as their impact on patient, family and provider experiences in diverse care settings.

## Author Contributions

**Alixe Ménard:** methodology, validation, visualisation, writing – review and editing, formal analysis, project administration, data curation, writing – original draft, investigation. **Daniel Kobewka:** funding acquisition, supervision, resources, software, project administration, conceptualisation, investigation, methodology, validation, writing – review and editing. **Yamini Singh:** conceptualisation, methodology, data curation, formal analysis, writing – review and editing. **Christina Yin:** conceptualisation, writing – review and editing. **Sydney Ruller:** conceptualisation, methodology, writing – review and editing. **Lauren Konikoff:** writing – review and editing. **Jackie Kierulf:** writing – review and editing. **Carol Bennett:** writing – review and editing. **Douglas Manuel:** writing – review and editing. **Shane Kirkham:** writing – review and editing. All authors have read and agreed to the published version of the manuscript.

## Ethics Statement

The study received research ethics approval from the Ottawa Health Science Network Research Ethics Board (protocol # 20240464‐01H). All procedures involving human participants were performed in accordance with relevant guidelines and regulations, including Canada's Tri‐Council Policy Statement: Ethical Conduct for Research Involving Humans (TCPS‐2) and Declaration of Helsinki.

## Consent

Informed consent for participation in the study was obtained by a qualitative researcher who explained the study, provided written information and answered any questions prior to obtaining verbal consent.

## Conflicts of Interest

The authors declare no conflicts of interest.

## Data Availability

The data that support the findings of this study are available from the primary author, Alixe Ménard (alimenard@ohri.ca), upon reasonable request.

## Appendix 1 Semi‐structured interview guide

**Table:**

Purpose	Question
Purpose and potential value of MyCare	1. Now that you have used MyCare, can you please tell me what the purpose of this tool might be? a. If unsure/no: How would MyCare be a useful tool for patients?
Perceived strengths and weaknesses of MyCare	b. What do you think was most useful about MyCare? c. Was there anything in MyCare that you found not useful?
User confidence	2. Did MyCare make you feel more confident in discussing your care preferences with others?
Perceived gap MyCare could fill	3. Let's think back to your last hospital visit when [repeat reason for visit]. Did you talk to your doctor about medical treatments you would or would not want? a. If yes: Were you asked about what mattered most to you in your health? (e.g., the ability to walk, to toilet yourself and to feed yourself). b. If unsure/no: Why not? i. Would it have been helpful to have been asked about your preferences related to treatments and about what mattered most to you in your health? 4. Are you familiar with the concept of ACP or future planning? [*ACP is making decisions about the healthcare you would want to receive if you became unable to communicate your wishes*] a. If yes: What do you know about it? i. Who explained this concept to you? b. If unsure/no: Do you think it would have been helpful for someone at the hospital to explain it to you?
Perceived usefulness of MyCare	5. How do you think MyCare could have been useful during your hospitalisation? a. How do you think it could have changed your experience?
Whether MyCare influenced the participant's preferences regarding specific medical treatments or interventions	6. Having now gone through MyCare, are there any medical treatments or interventions you would not want? a. Did MyCare help you think about this [or were you already thinking about this?]
The perceived impact of MyCare on a patient's confidence in communicating their care preferences	7. Imagine a patient didn't know about ACP. Do you think MyCare could change their confidence [be it positively or negatively] in communicating with their health team about their preferences for care and what matters to them most? a. How so? [Can you please elaborate?]
Whether MyCare prompted reflection on the participant's health and future healthcare decisions	8. Did MyCare prompt you to reflect on your health and consider decisions regarding your healthcare, either now or for the future? a. If yes: Can you please walk me through one of these reflections?
Exploring the participant's personal values and criteria for an acceptable quality of life	9. When using MyCare, you were prompted to think about what an acceptable quality of life looks like to you. Can you please walk me through what you believe to be an acceptable quality of life [for you]?
The perceived usefulness of MyCare in preparing patients for making medical treatment decisions	10. Do you think MyCare might be able to help patients prepare for medical treatment decisions? a. How so? [Can you please elaborate?]
The potential role of MyCare in assisting patients with the selection of a substitute decision‐maker	11. Do you think MyCare can help patients pick a substitute decision‐maker if they were to become too sick to make decisions for themselves? a. How so? [Can you please elaborate?]
The potential of MyCare to facilitate communication between patients and their doctors regarding the patients' priorities and wishes	12. Do you think MyCare can help patients talk to their doctors about what is most important to them so that their wishes are respected? a. How so? [Can you please elaborate?]
Exploring the need for comprehensive information within MyCare to support informed decision‐making regarding personal healthcare choices	13. Is there any information you wish had been included in MyCare that could help you make more informed decisions about your care? [Reframe: Was there something missing from the tool?] a. How would this [insert their recommendation] help you?
Participants' emotional responses and experiences while using MyCare	14. How did you feel while you were navigating through the tool? a. Was there anything that made it difficult or easy for you to navigate? b. Was there anything surprising about your experience using MyCare that you didn't expect to come up? [e.g., emotions due to difficult topics, thinking about states worse than death and so forth]
N/A	15. Is there anything else you would like to share about your experience with MyCare today?

## Appendix 2 Codebook for thematic analysis

**Table:**

Code	Definition
MyCare‐Purpose	Purpose and potential value of MyCare from a user's perspective.
MyCare‐Weakness	Perceived weaknesses of MyCare or perceived challenges in its use by patients or care partners.
MyCare‐Strength	Perceived strengths of MyCare.
MyCare‐Confidence	MyCare's ability to increase confidence in discussing care preferences with others.
MyCare‐Gap	Perceived gaps MyCare could fill.
MyCare‐Delivery	Any mention of how to deliver MyCare to prospective patients and who is the best recipient (e.g., patients vs. caregivers).
MyCare‐Edits	Proposed edits to MyCare by participants.
ACP or Future Planning	Any mention of ACP or future planning relevant to healthcare or living. Participant's familiarity or knowledge of the concept of ACP/FP. Who explained ACP/FP or provided information. Whether participants thought ACP/FP should have been explained in the hospital.
Care preferences	Whether the participant discussed treatments or health priorities with their doctor.
Barriers to communication	Reasons participants did not discuss preferences with healthcare providers.
Reflection on treatment choices	Whether MyCare prompted thoughts about treatments/care choices the participant would or would not want (including long‐term care).
Acceptable quality of life	Participant's explanation of what they consider an acceptable quality of life + treatments/care options they would consider acceptable.
Unacceptable quality of life	Participant's explanation of what they consider an unacceptable quality of life + treatments/care options they would consider unacceptable (including long‐term care).
Impact on family or friends	Any mention of their illness or future health state negatively impacting their family or friends.
Family doctor	Any mention of the participant's family doctor or lack thereof.
SDM	Whether MyCare helps participants identify a substitute decision‐maker or a power of attorney. Any mention of SDM or POA.
Emotional impact	How the tool affected participants emotionally (e.g., stress, sadness and relief).
Past experience	Any mention of a past experience and its influence on decision‐making or future planning (e.g., their mother's experience in LTC and their parent's stroke).

## Appendix 3 Summary of electronic health assessment, system usability scale and theoretical framework of acceptability

**Table:**

Electronic Health Assessment		
Question	Median	IQR
How familiar are you with a computer keyboard?	4	2
How familiar are you with computer settings?	3	3
How familiar are you with a computer's operating system?	2	2
How familiar are you with the concept of a username?	3	3
How confident do you feel about using a computer in general?	3	1
How confident do you feel about using a touchscreen?	3	2
How confident do you feel in finding information online?	3	1
How much do you enjoy using a computer in general?	3	3
How much do you enjoy using a touchscreen?	3	3
How much do you enjoy finding information online?	4	3
**System Usability Scale**		
I think that I would like to use this system frequently.	4	2
I found the system unnecessarily complex.	1	1
I thought the system was easy to use.	5	1
I think that I would need the support of a technical person to be able to use this system.	2	3
I found the various functions in this system were well integrated.	4	2
I thought there was too much inconsistency in this system.	2	2
I would imagine that most people would learn to use this system very quickly.	5	1
I found the system very cumbersome to use.	1	1
I felt very confident using the system.	5	1
I needed to learn a lot of things before I could get going with this system.	1	1
**Theoretical Framework of Acceptability**		
How comfortable did you feel when you were completing MyCare?	5	1
How much effort did it take to complete MyCare?	1	1
There are moral or ethical consequences with MyCare.	1	2
MyCare has improved my understanding of my health wishes and goals.	4	3
It is clear to me how MyCare will help me to engage in my care planning.	5	1
How confident did you feel about completing MyCare by yourself?	5	1
MyCare interfered with my other priorities.	1	1
How acceptable is MyCare to you?	5	1
